# Pioglitazone Reduces Hepatocellular Carcinoma Development in Two Rodent Models of Cirrhosis

**DOI:** 10.1007/s11605-018-4004-6

**Published:** 2018-10-26

**Authors:** Shen Li, Sarani Ghoshal, Mozhdeh Sojoodi, Gunisha Arora, Ricard Masia, Derek J. Erstad, Michael Lanuti, Yujin Hoshida, Thomas F. Baumert, Kenneth K. Tanabe, Bryan C. Fuchs

**Affiliations:** 1 Division of Surgical Oncology, Massachusetts General Hospital Cancer Center and Harvard Medical School, 55 Fruit Street, WRN 401, Boston, MA 02114 USA; 2 Department of Pathology, Massachusetts General Hospital, Harvard Medical School, Boston, MA USA; 3 Liver Tumor Translational Research Program, Harold C. Simmons Comprehensive Cancer Center, Division of Digestive and Liver Diseases, Department of Internal Medicine, University of Texas Southwestern Medical Center, Dallas, TX USA; 4 Inserm, U1110, Institut de Recherche sur les Maladies Virales et Hépatiques, Strasbourg, France

**Keywords:** PPARγ, HCC, Chemoprevention, Fibrosis, AMPK, NASH

## Abstract

**Background:**

Hepatocellular carcinoma (HCC) is one of the deadliest malignancies worldwide due to the lack of effective treatments. Chemoprevention in high-risk patients is a promising, alternative strategy. In this study, pioglitazone was investigated for its ability to prevent hepatocarcinogenesis in two rodent models of cirrhosis.

**Methods:**

In the first model, male Wistar rats were given repeated, low-dose injections of diethylnitrosamine (DEN) to accurately recapitulate the progression of fibrosis to cirrhosis and HCC. In the second model, a single dose of DEN was administered to male C57Bl/6 pups at day fifteen followed by administration of a choline-deficient, L-amino acid defined, high-fat diet (CDAHFD) at week six for 24 weeks. Pioglitazone treatment started at the first signs of fibrosis in both models.

**Results:**

Pioglitazone effectively reduced fibrosis progression and HCC development in both models. Gross tumor nodules were significantly reduced after pioglitazone treatment (7.4 ± 1.6 vs. 16.6 ± 2.6 in the rat DEN model and 5.86 ± 1.82 vs. 13.2 ± 1.25 in the mouse DEN+CDAHFD model). In both models, pioglitazone reduced the activation of mitogen-activated protein kinase (MAPK) and upregulated the hepato-protective AMP-activated protein kinase (AMPK) pathway via increasing circulating adiponectin production.

**Conclusion:**

Pioglitazone is an effective agent for chemoprevention in rodents and could be repurposed as a multi-targeted drug for delaying liver fibrosis and hepatocarcinogenesis.

**Electronic supplementary material:**

The online version of this article (10.1007/s11605-018-4004-6) contains supplementary material, which is available to authorized users.

## Introduction

Hepatocellular carcinoma (HCC) is the fifth most common cause of cancer, and the second leading cause of cancer-related deaths globally.^1^ Given the growing number of patients with chronic hepatitis C and the increasing risk of non-alcoholic fatty liver disease (NAFLD) as a result of the obesity epidemic, the incidence of HCC will continue to rise over the next few decades.^2, 3^ The 5-year survival rate for patients with HCC is abysmal and only 13% of diagnosed patients undergo a curative resection.^4^ Given the high mortality rate associated with HCC as well as the readily available cohort of patients at risk for HCC development, specifically those with HBV, HCV, diabetes, and NAFLD, effective chemoprevention strategies could help improve survival by preventing or delaying the onset of cancer.

The ideal chemoprevention agent should be safe and readily available. There is growing evidence that anti-diabetic medications, such as metformin, are effective in halting HCC carcinogenesis in both in vitro and in vivo settings.^5, 6^ Thiazolidinediones (TZDs) including pioglitazone, troglitazone, and rosiglitazone are another major family of anti-diabetic medications and have also been purported to be anti-carcinogenic. TZDs are high-affinity ligands for the nuclear receptor peroxisome proliferator-activated receptor gamma (PPARγ).^7^ TZDs function to improve insulin sensitivity by regulating glucose homeostasis and fatty acid metabolism. The PIVENS trial demonstrated that pioglitazone treatment significantly reduced steatosis and inflammatory features such as hepatocellular ballooning in non-alcoholic steatohepatitis (NASH) patients.^8^ PPARγ agonists have also been shown to decrease inflammation and tumorigenesis in multiple tumor models, such as colon, thyroid and lung cancers.^9^

However, the role of PPARγ agonists in preventing HCC development is less clear. Yu et al. found an increased tumor burden in PPARγ deficient mice in a diethylnitrosamine-induced HCC model, while prophylactic treatment with rosiglitazone decreased the incidence of HCC.^10^ In addition, a major risk factor for HCC development is ongoing fibrogenesis leading to cirrhosis, ultimately predisposing hepatocytes to malignant transformation. Pioglitazone and rosiglitazone have been shown to reduce in vitro activation of hepatic stellate cells, the cells responsible for extracellular matrix (ECM) deposition during hepatic fibrosis.^11, 12^ Despite pre-clinical data demonstrating a negative correlation between PPARγ expression level and tumorigenesis, few studies have evaluated the direct effects of TZDs on the fibrosis/cirrhosis/carcinogenesis axis in the liver.

Therefore, in the following study, we explored the effects of one of the most commonly prescribed TZDs, pioglitazone, on fibrosis progression and HCC development in two rodent models of cirrhosis.

## Material and Methods

### Chemicals

Pioglitazone was freshly dissolved in 0.5% methylcellulose before administration.

### Animal Experiments

Animal experiments were approved by the Massachusetts General Hospital Institutional Animal Care and Use Committee and all animals received humane care according to the criteria outlined in the *Guide for the Care and Use of Laboratory Animals of the National Academy of Sciences*. Male Wistar rats (Charles River Laboratories, Wilmington, MA) were subjected to either weekly intraperitoneal injections of PBS (*n* = 6) or 50 mg/kg diethylnitrosamine (DEN) (*n* = 18) (Sigma, St. Louis, MO) for 18 weeks. DEN is an environmental carcinogen that produces mutagenic DNA adducts, leading to gene sequence mismatches, ultimately predisposing the liver to HCC formation.^13^ Based on our prior studies, repeated, low-dose DEN administration accurately models chronic liver disease progression in humans with development of fibrosis at 8 weeks, early cirrhosis at 12 weeks, and fulminant cirrhosis and carcinogenesis at 18 weeks.^14^ After 8 weeks of injections, DEN-injured rats began treatment via oral gavage with pioglitazone 3 mg/kg or vehicle (0.5% methylcellulose) for the remainder of the study (*n* = 9 for each group).

A single injection of DEN to developing mice on day 15 is a common method to generate HCCs in 9–12 months although usually in the setting of a normal liver. Recently, a choline-deficient, L-amino acid-defined, high-fat diet consisting of 60 kcal% fat and 0.1% methionine by weight (CDAHFD) has been shown to recapitulate features of NASH.^15^ We combined these approaches to accelerate the process of carcinogenesis in the setting of a NASH cirrhosis background from 52 weeks in CDAHFD alone^16^ to 24 weeks with DEN + CDAHFD. Thus, male C57Bl/6 mice (Charles River Laboratories) received a single dose of 35 mg/kg DEN at day 15. At 6 weeks of age, mice were subjected to either standard chow (*n* = 5) or CDAHFD (*n* = 16) for a total of 24 weeks. Oral gavage of either vehicle control (*n* = 8) or pioglitazone 10 mg/kg (n = 8) was initiated 6 weeks following the onset of CDAHFD for the remainder of the study.

A human pioglitazone dose of 45 mg per day was converted to a rat dose of 3 mg/kg and a mouse dose of 10 mg/kg based on a body surface area calculation. For both models, animals were anesthetized and sedated at the time of sacrifice. A terminal blood collection was performed by cardiac puncture. Livers were weighed, fixed in formalin, and snap frozen for further analysis.

### Serum Laboratory Analysis

Blood was allowed to clot for 1 h at room temperature before centrifugation at 2000 rpm for 10 min at 4 °C. Serum was isolated and stored at − 80 °C prior to use. Serum levels of alkaline phosphatase (ALP), aspartate transaminase (AST), alanine aminotransferase (ALT), total bilirubin (TBIL), and Gamma-glutamyl transferase (GGT) were measured.

### Histology, Immunohistochemistry, Immunofluorescence

Formalin-fixed samples were embedded in paraffin, cut in 5 μm thick sections and stained with hematoxylin & eosin (H&E) or Sirius red. The collagen proportional area (CPA) was morphometrically quantified on Sirius red-stained sections with image processing software (Image J, NIH). All slides were blindly reviewed by an independent liver pathologist to calculate the NAFLD activity score (NAS) and the fibrosis score. Additional sections in the DEN model were stained with an antibody specific for proliferating cell nuclear antigen (PCNA) (Cell Signaling Technology, Danvers, MA). For immunofluorescence, sections were stained for α-SMA (Abcam, Cambridge, MA) with detection by appropriate secondary antibodies labeled with either Cy3 or Alexa488 according to the manufacturer’s instructions.

### Western Blotting

Livers were homogenized and protein was extracted using radioimmunoprecipitation assay buffer (RIPA, Boston BioProducts, Ashland, MA) containing phosphatase and protease inhibitors (Sigma, St. Louis, MO). Protein concentrations were normalized to 100 μg using the bicinchoninic acid (BCA) method (Pierce Chemical Co., Rockford, IL). Protein samples were separated based on molecular weight and transferred to a polyvinylidene difluoride membrane (Millipore, Billerica, MA). Membranes were incubated with primary antibodies specific for total 5′ adenosine monophosphate-activated protein kinase (AMPK), phosphorylated (Thr172) AMPK, total acetyl-coA carboxylase (ACC), phosphorylated (Ser79) ACC, total p44/42 mitogen-activated protein kinase MAPK (Erk1/2), phosphorylated (Thr202/Tyr204) Erk1/2, total c-Jun N-terminal kinase (SAPK/JNK), phosphorylated (Thr183/Tyr185) SAPK/JNK, total c-Jun, phosphorylated (Ser73) c-Jun, total p38 mitogen-activated protein kinase (P38), and phosphorylated (Thr180/Tyr182) P38 (all antibodies from Cell Signaling Technology). β-actin (Abcam, Cambridge, MA) was used as a loading control. Blots were incubated with appropriate secondary antibodies conjugated to horseradish peroxidase (HRP; GE Healthcare, United Kingdom). Western blots were repeated at least twice to ensure reproducibility.

### ELISA

Adiponectin protein expression was quantified in serum using a commercially available ELISA (Millipore, Burlington, MA) according to the manufacturer’s instructions. Each sample was quantified in duplicate and the experiments were repeated in both animal models to ensure accuracy and reproducibility. 8-Hydroxydeoxyguanosine (8-OHdG) levels were quantified in tissue using a commercially available ELISA system (Cell Biolabs, San Diego, CA).

### RNA Isolation and Reverse Transcription

RNA was isolated from liver tissue using TRIzol (Life Technologies, Grand Island, NY) according to the manufacturer’s instructions and then treated with DNAse I (Promega, Madison, WI). Total RNA (1 μg) from each sample was used to synthesize complementary DNA by single-strand reverse transcription (SuperScript III First-Strand Synthesis SuperMix; Life Technologies). Expression of actin, alpha 2, smooth muscle, aorta (*Acta2*), collagen, Type 1, alpha 1 (*Col1a1*), chemokine (C-C motif) ligand 2 (*Ccl2*), cluster of differentiation 68 (*Cd68*), interferon-gamma (*Ifn-γ*), interleukin 6 (*Il6*), TIMP metallopeptidase inhibitor I (*Timp1*), and transforming growth factor beta 1 (*Tgf-β1*) were analyzed by quantitative real-time PCR using TaqMan gene expression assays (Thermo Fisher Scientific, Waltham, MA) on Applied Bioscience 7900HT Fast Real-Time PCR system using 384 well plates with a reaction volume of 10 μl. The 2−ΔCT method was used for relative quantification of mRNA with normalization to 18S. Taqman probe sets used were: 18S (Hs03003631_g1), *Acta2* (Mm00725412_g1 and Rn01759928_g1), *Col1a1* (Mm00801666_g1 and Rn01463848_m1), *Ccl2* (Mm00441242_m1), *Cd68* (Mm03047343_m1), Ifn-γ (Mm01168134_m1), *Il6*(Mm00446190_m1), *Timp1* (Mm01341361_m1 and Rn01430874_g1), and *Tgf-β1* (Mm01178820_m1 and Rn00572010_m1).

### Statistical Analysis

Data are represented as mean ± standard deviation. An unpaired two-tailed *t* test was used to compare differences between groups.

## Results

### Pioglitazone Reduces Carcinogenesis in the DEN Rat Model

**Fig. 1:**
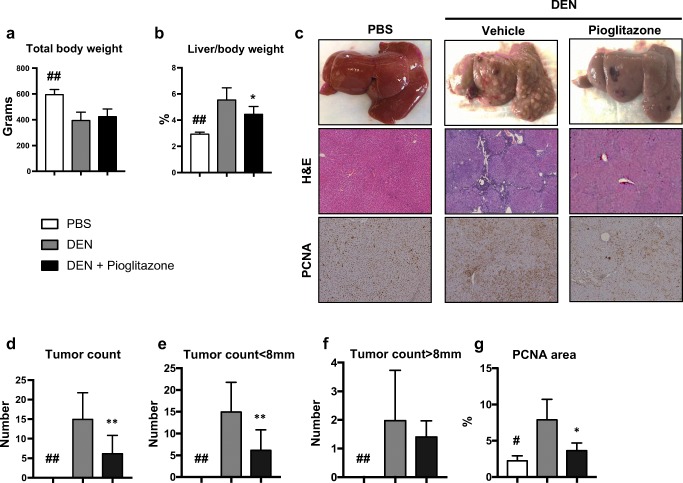
Pioglitazone reduces carcinogenesis in the rat DEN model. At the time of sacrifice, **a** overall body weight and **b** liver weight/body weight ratios were measured. **c** Representative gross pictures (top panel), representative H&E staining (× 5 original magnification, middle panel), and immunohistochemical proliferating cell nuclear antigen (PCNA) staining (× 5 original magnification, bottom panel) are shown. **d** Total surface tumors were counted and separated by size as **e** < 8 mm and **f** > 8 mm. **g** PCNA staining was quantified using image J software. # *p* < 0.05 and ## *p* < 0.01 compared to PBS. * *p* < 0.05 and ** *p* < 0.01 compared to DEN

Weekly administration of DEN to rats resulted in a significant reduction in body weight (398.1 ± 22.7 vs. 598.6 ± 20.73; *p* < 0.01), and an increase in the liver/body weight ratio (5.6 ± 0.34 vs. 2.9 ± 0.06; *p* < 0.01) as a result of increased tumor burden. Pioglitazone administration led to an improved body weight (427.5 ± 18.7 vs. 398.1 ± 22.7) and a significant reduction in liver body weight ratio (4.5 ± 0.21 vs. 5.6 ± 0.34; *p* < 0.05) (Fig. 1a, b). Repeated DEN administration also predictably resulted in liver failure as measured by liver function tests, and pioglitazone treatment led to a significant improvement in total bilirubin (1.2 ± 0.42 vs. 3.6 ± 0.83; *p* < 0.05) (Supplementary Fig. 1a-e).

Macroscopically, the livers from rats treated with the PBS vehicle control appeared normal, while livers from DEN-injured rats were cirrhotic and contained many HCCs (Fig. 1c top panel). Importantly, pioglitazone treatment resulted in a 55% reduction in the number of gross tumor nodules (7.4 ± 1.6 vs. 16.6 ± 2.6; *p* < 0.01) (Fig. 1d). Specifically, pioglitazone treatment significantly reduced the appearance of small (< 8 mm) tumor nodules (15.1 ± 2.5 vs. 6.3 ± 1.5; *p* < 0.01) (Fig. 1e), but did not have a significant effect on the number of large (> 8 mm) tumor nodules (Fig. 1f). Microscopically, H&E slides from DEN-injured rats contained tumors as well as peri-tumoral extracellular matrix deposition (Fig. 1c middle panel). Proliferating cell nuclear antigen (PCNA) is a marker for active cellular proliferation.^17^ DEN-injured rats had increased PCNA staining in comparison to pioglitazone treated rats (8 ± 1.4 vs. 3.8 ± 0.5; *p* < 0.5) (Fig. 1c bottom panel and Fig. 1g). The anti-tumor effects of pioglitazone were not attributable to a drug interaction or alterations in DEN metabolism, as liver levels of 8-hydroxydeoxyguanosine (8-OHdG), a marker of oxidative stress-derived DNA damage often used as a surrogate for DEN activity,^18^ were similar between DEN-injured rats treated with vehicle control or pioglitazone (0.22 ± 0.02 vs. 0.22 ± 0.02) (Supplementary Fig. 2).

### Pioglitazone Reduces Fibrosis/Cirrhosis in the Rat DEN Model

**Fig. 2:**
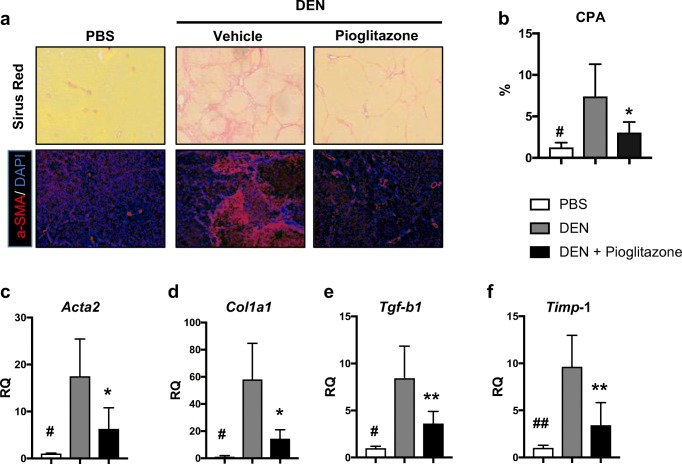
Pioglitazone reduces fibrosis/cirrhosis in the rat DEN model. **a** Sirius red staining (× 10 original magnification, top panel) and α-SMA immunofluorescence staining (× 10 original magnification, bottom panel) were performed to assess fibrosis and hepatic stellate cell activation, respectively. **b** Morphometric assessment of the collagen proportional area (CPA) on sirius red-stained slides. Gene expression of fibrosis markers including **c***Acta2*, **d***Col1a1*, **e***Tgf-b1*, and **f***Timp1*. # *p* < 0.05 and ## *p* < 0.01compared to PBS. * *p* < 0.05 and ** *p* < 0.01 compared to DEN

DEN-injured rats appeared cirrhotic grossly, while livers from pioglitazone treatment were less fibrotic. Fibrosis was further assessed using Sirius red staining. In DEN-injured rats, nodular cirrhosis was visually evident (Fig. 2a top panel). Morphometric quantification of the collagen proportional area (CPA) demonstrated a marked increase in collagen deposition (7.4 ± 1.5 vs. 1.3 ± 0.3; *p* < 0.05) in DEN-injured rats as compared to PBS controls. Although bridging fibrosis was still observed after pioglitazone treatment, the CPA was significantly decreased in comparison to DEN-injury alone (3.1 ± 0.45 vs 7.4 ± 1.5; *p* < 0.05) (Fig. 2b). In addition, immunofluorescence staining of alpha-smooth muscle actin (α-SMA) demonstrated a reduction in hepatic stellate cell activation after pioglitazone treatment (Fig. 2a bottom panel). Finally, pioglitazone decreased the mRNA expression of several well-established pro-fibrotic markers in comparison to DEN-injury alone including *Acta2* (RQ = 3.7 ± 1.7 vs. 17.5 ± 3.7; *p* < 0.05), *Col1a1* (RQ = 6.3 ± 1.7 vs. 17.5 ± 3.5; *p* < 0.05), *Tgf-b1* (RQ = 3.6 ± 0.5 vs. 8.4 ± 1.5; *p* < 0.01), and *Timp1* (RQ = 3.4 ± 0.9 vs. 9.6 ± 1.5; *p* < 0.01) (Fig. 2c–f).

### Pioglitazone Reduces Proliferation via Activation of the AMPK Pathway and Downregulation of the MAPK Pathway

**Fig. 3:**
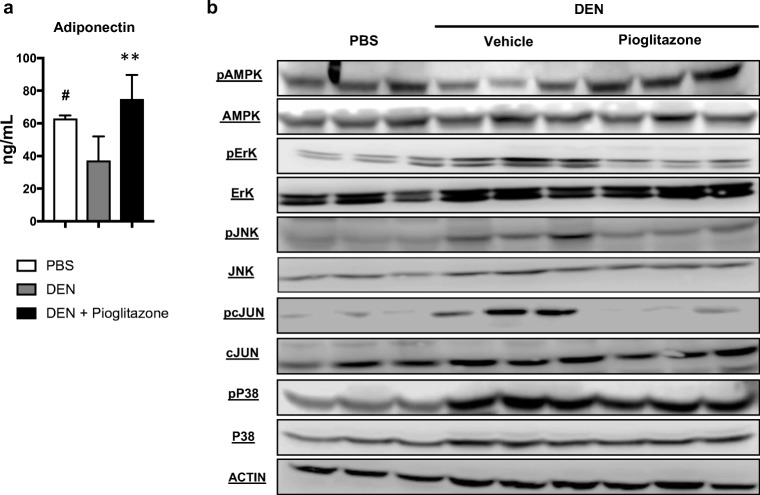
Pioglitazone increases serum adiponectin and liver AMPK activation and decreases liver MAPK signaling in the rat DEN model. **a** Serum adiponectin was measured in the rat DEN model. **b** Western blot analysis of phosphorylated (Thr172) 5′ adenosine monophosphate-activated protein kinase (pAMPK)/total AMPK, phosphorylated (Thr202/Tyr204)-p44/42 mitogen-activated protein kinase MAPK (pErk1/2)/ total Erk1/2, phosphorylated (Thr183/Tyr185) c-Jun N-terminal kinase (pSAPK/JNK)/total SAPK/JNK, phosphorylated (Ser73) c-Jun/total c-Jun, phosphorylated (Thr180/Tyr182) p38 mitogen-activated protein kinase (pP38)/total P38. Actin was used as a loading control. # *p* < 0.05 and ## *p* < 0.01 compared to PBS. * *p* < 0.05 and ** *p* < 0.01 compared to DEN

Serum adiponectin levels are known to decrease in patients with cirrhosis and HCC as compared to healthy controls.^19^ We similarly observed decreased serum adiponectin levels in DEN-injured rats as compared to healthy PBS animals (37.3 ± 7 vs 62.9 ± 1.1; *p* < 0.05). Treatment with pioglitazone normalized serum adiponectin levels (74.8 ± 5.63 vs. 37.3 ± 7.3; *p* < 0.01) (Fig. 3a). Adiponectin is known to activate AMP-activated protein kinase (AMPK), a serine/threonine protein kinase that regulates cellular energy homeostasis.^20^ Activated AMPK can act as a tumor suppressor by directly inducing cell-cycle arrest.^21^ We observed decreased phosphorylated AMPK in the livers of DEN-injured rats as compared to livers from PBS animals. Pioglitazone treatment normalized liver levels of phosphorylated AMPK similar to PBS animals. In addition, treatment with pioglitazone reduced multiple mitogen-activated protein kinase (MAPK) pathways, including reductions in phosphorylated extracellular signal-regulated kinase (pERK), c-JUN N-terminal kinase (pJNK), and its downstream target phosphorylated c-JUN. By comparison, no changes in the level of phosphorylated-P38 MAPK were observed between the groups (Fig. 3b).

### Pioglitazone Reduces Carcinogenesis in the Mouse DEN+CDAHFD Model

**Fig. 4:**
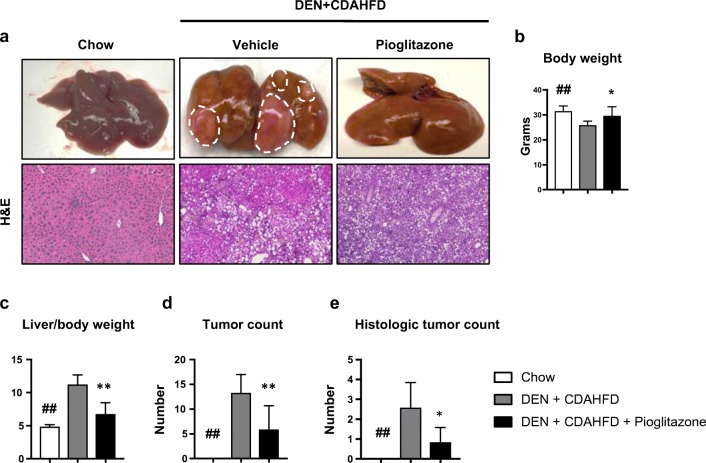
Pioglitazone reduces carcinogenesis in the mouse DEN + CDAHFD model. **a** Representative gross pictures of livers (top panel) and H&E staining (× 5 original magnification, bottom panel). White dotted lines highlight macroscopic tumor nodules. **b** Body weight and **c** liver/body weight ratio were measured at the time of sacrifice. **d** Surface tumors > 1 mm were counted. **e** HCCs were counted on whole scanned H&E sections of the entire left liver by a liver pathologist. # *p* < 0.05 and ## *p* < 0.01 compared to standard chow. * *p* < 0.05 and ** *p* < 0.01 compared to DEN+CDAHFD

We next sought to examine the effects of pioglitazone on NASH-driven HCC. Male mice given a single injection of DEN on day 15 followed by feeding of CDAHFD on week 6 for a total of 24 weeks (DEN+CDAHFD) developed HCC with 100% incidence (Fig. 4a top panel). Pioglitazone use in humans is associated with weight gain and we similarly observed a significant increase in total body weight in DEN+CDAHFD mice treated with pioglitazone as compared to vehicle control (29.3 ± 1.4 vs. 25.963 ± 0.55; *p* < 0.05) (Fig. 4b). The combination of DEN+CDAHFD resulted in lipid laden HCCs or HCC nodules surrounded by fatty deposition (Fig. 4a bottom panel). Liver/body weight ratio was used as a tumor burden surrogate. As compared to DEN+CDAHFD mice treated with vehicle, pioglitazone treatment decreased the liver/body weight ratio (6.75 ± 0.65 vs. 11.22 ± 0.49; *p* < 0.01). Grossly, we measured all tumors greater than 1 mm, and found that pioglitazone treatment resulted in a significant reduction in the number of surface tumors (5.86 ± 1.82 vs. 13.2 ± 1.25; *p* < 0.01). In addition, the entire left lobe of each animal was formalin-fixed and H&E-stained for analysis of microscopic nodules by a blinded liver pathologist. Pioglitazone treatment resulted in a significant reduction in histological tumor nodules as well (0.83 ± 0.31 vs 2.6 ± 0.5; *p* < 0.05) (Fig. 4c–e).

### Pathological Assessment of the Mouse DEN+CDAHFD Model

**Fig. 5:**
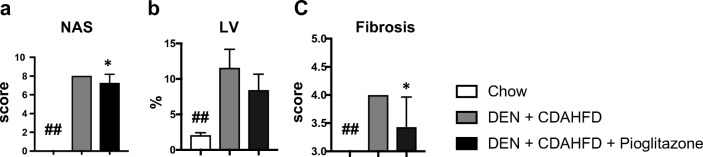
Pioglitazone reduces pathological scoring in the mouse DEN + CDAHFD model. **a** The non-alcoholic fatty liver disease (NAFLD) activity score (NAS) and **b** the NASH fibrosis score were scored by a blinded liver pathologist. **c** Lipid vacuolization (LV) was morphometrically calculated using image J software. ## *p* < 0.01 compared to standard chow. * *p* < 0.05 compared to DEN+CDAHFD

The NAFLD activity score (NAS) (7.3 ± 0.48 vs 8 ± 0; *p* < 0.05) and fibrosis scores (3.5 ± 0.22 vs 4 ± 0; *p* < 0.05) were significantly reduced with pioglitazone treatment. The NAS score comprises steatosis, lobular inflammation as well as hepatocyte ballooning. Notably, pioglitazone decreased steatosis without reducing inflammation or hepatocyte ballooning. To verify this former finding, the area of lipid vacuolization was morphometrically measured and a significant reduction of fat deposition was seen with pioglitazone administration (8.4 ± 0.84 vs. 11.6 vs. 0.9; *p* < 0.08), consistent with the reduced steatosis observed within the NAS scoring criteria (Fig. 5a–c).

### Pioglitazone Reduces Inflammation and Fibrosis in the Mouse DEN+CDAHFD Model

**Fig. 6:**
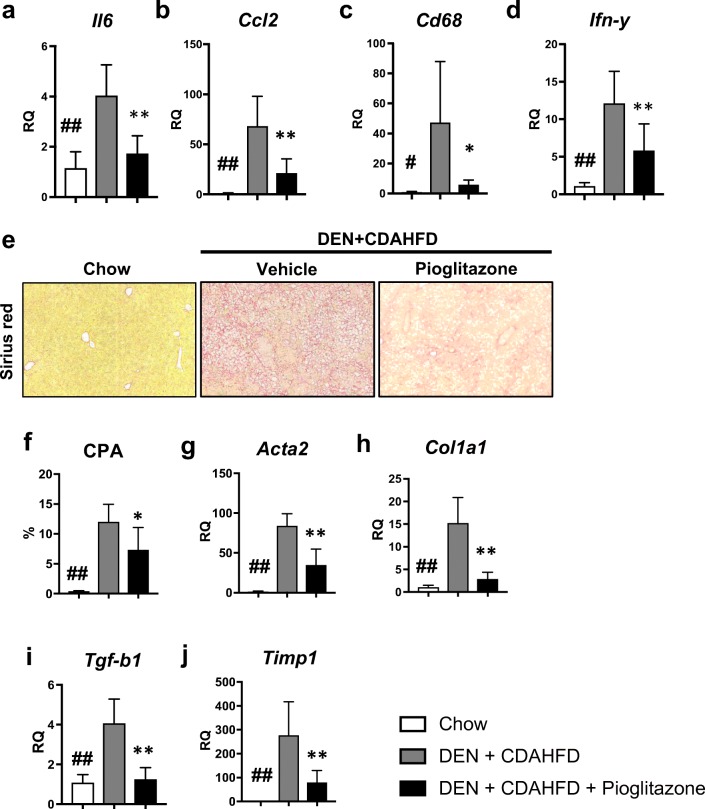
Pioglitazone reduces inflammation and fibrosis in the mouse DEN + CDAHFD model. Gene expression of several pro-inflammatory markers including **a***Il6*, **b***Ccl2*, **c***Cd68*, and **d***Ifn-y* was measured. **e** Sirius red staining was performed to assess fibrosis and the **f** collagen proportional area (CPA) was calculated. Fibrotic gene expression including **g***Acta2*, **h***Col1a1*, **i***Tgfb-1*, and **j***Timp1* was measured. **k** The non-alcoholic fatty liver disease (NAFLD) activity score (NAS) and **l** the NASH fibrosis score were scored by a blinded liver pathologist. **m** Lipid vacuolization (LV) was morphometrically calculated using image J software. # *p* < 0.05 and ## *p* < 0.01 compared to standard chow. * *p* < 0.05 and ** *p* < 0.01 compared to DEN+CDAHFD

While no differences were seen on histological scoring of inflammation, this score does not distinguish between inflammatory cell types. To examine inflammation more closely, we examined the expression of several pro-inflammatory genes. We observed a significant reduction in *Il6* (RQ = 1.7 ± 0.27 vs. 4.0 ± 0.46; *p* < 0.01), *Ccl2* (RQ = 21.4 ± 5.4 vs. 68.2 ± 11.2; *p* < 0.01), *Cd68* (RQ = 6.0 ± 1.1 vs. 47.3 ± 15.4; *p* < 0.05), and *Ifn-y* (RQ = 5.8 ± 1.3 vs. 12.1 ± 1.6; *p* < 0.01) (Fig. 6a–d).

Similar to the repeated, low-dose rat DEN cirrhosis model, we also noted a decrease in hepatic fibrosis with pioglitazone treatment. The CPA was significantly reduced with pioglitazone treatment (7.3 ± 1.5 vs. 11.9 ± 0.98; *p* < 0.05) (Fig. 6e, f). We also measured the mRNA expression of *Acta2 (*(RQ = 2.9 ± 0.6 vs. 15.2 ± 2.1; *p* < 0.01)*, Col1a1* (RQ = 34.9 ± 7.5 vs. 83.9 ± 5.8; *p* < 0.01), *Tgf-b1* (RQ = 1.2 ± 0.2 vs 4.1 ± 0.5; *p* < 0.01)*, and Timp1* (RQ = 79.3 ± 18.7 vs. 277 ± 52.9; *p* < 0.01) (Fig. 6g–j) and found a significant reduction in all four pro-fibrotic markers with pioglitazone treatment.

### Pioglitazone Increases AMPK Activation and Decreases MAPK Signaling

**Fig. 7:**
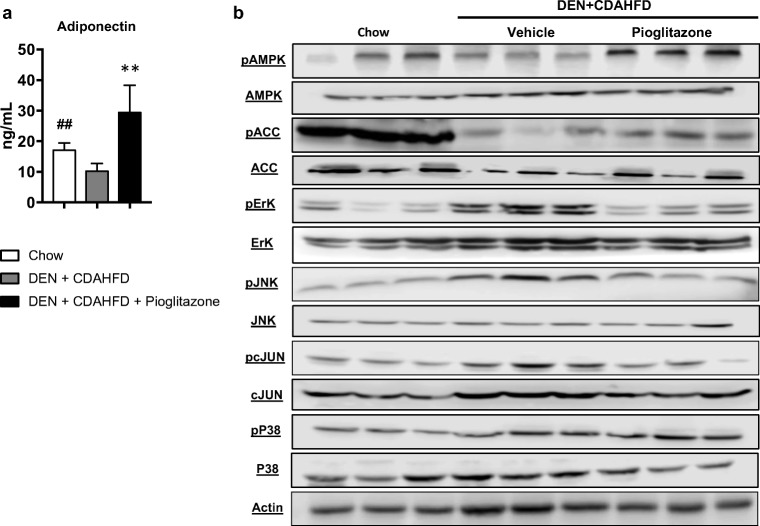
Pioglitazone increases serum adiponectin and liver AMPK activation and decreases liver MAPK signaling in the mouse DEN + CDAHFD model. **a** Serum adiponectin was measured in the mouse DEN+CDAHFD model. **b** Western blot analysis of phosphorylated (Ser79) Acetyl-CoA Carboxylase (pACC)/total ACC, phosphorylated (Thr172) 5′ adenosine monophosphate-activated protein kinase (pAMPK)/total AMPK, phosphorylated (Thr202/Tyr204)-p44/42 mitogen-activated protein kinase MAPK (pErk1/2)/ total Erk1/2, phosphorylated (Thr183/Tyr185) c-Jun N-terminal kinase (pSAPK/JNK)/ total SAPK/JNK, phosphorylated (Ser73) c-Jun/total c-Jun, phosphorylated (Thr180/Tyr182) p38 mitogen-activated protein kinase (pP38)/total P38. Actin was used as a loading control. # *p* < 0.05 and ## *p* < 0.01 compared to PBS or standard chow. * *p* < 0.05 and ** *p* < 0.01 compared to DEN or DEN+CDAHFD

In this NASH model of HCC, we observed a significant reduction in serum adiponectin levels as compared to mice fed standard chow (10.5 ± 0.79 vs. 17.3 ± 0.94; *p* < 0.01), and pioglitazone administration increased circulating serum adiponectin in DEN+CDAHFD mice (29.7 ± 3.2 vs. 10.5 ± 0.79; *p* < 0.01) (Fig. 7a). In addition, DEN+CDAHFD mice treated with pioglitazone had increased activation of AMPK, as well as decreased activation of ERK, JNK, and its downstream target c-JUN. As observed in the rat DEN model, no changes in the level of phosphorylated P38 were seen between groups in the DEN+CDAHFD model as well. Lastly, activated AMPK phosphorylates acetyl coA carboxylase (ACC), thus inactivating this rate-limiting step for fatty acid synthesis. Pioglitazone treatment increased phosphorylated ACC and this may have resulted in the observed decrease in steatosis seen within the NAS scoring criteria as well as the lipid vacuolization quantification (Fig. 7b).

## Discussion

Underlying cirrhosis is associated with 80–90% of patients with primary HCC^22^ and thus at risk patients are easily identifiable unlike many other malignancies. With the rising incidence of obesity and diabetes, NAFLD/NASH-related hepatic fibrosis/cirrhosis will likely become the most common cause of HCC in the future.^23^ Thus, there is increased interest in the use of easily accessible and inexpensive medications, like anti-diabetic drugs, as chemopreventive strategies.

In this study, the administration of pioglitazone at the onset of fibrosis in both animal models resembles “primary chemoprevention”, the administration of an agent to patients without overt disease but with known risk factors.^24^ The low-dose, repeated DEN rat model was used given its similarity at the histologic and transcriptomic level to human cirrhosis.^25^ We observed a significant reduction in tumor nodules in the rat DEN model after treatment with pioglitazone. This effect was specific to smaller nodules (< 8 mm) suggesting that pioglitazone prevented the development of new HCCs, but had no effect on the growth of established tumors. Given its use as an anti-diabetic medication, we also tested pioglitazone in a mouse NASH-HCC model. We also observed decreased tumor incidence when pioglitazone was used to treat mice subjected to a single dose of DEN followed by a long-term feeding of CDAHFD.

Another piece of evidence supporting the preventive effects of pioglitazone is the significant reduction of underlying fibrosis/cirrhosis. The pathogenesis of HCC in a fibrotic/cirrhotic background is still unclear. The discrepancy lies in the unsettled question of whether fibrogenesis promotes HCC carcinogenesis or if the fibrosis is a byproduct of chronic inflammation and liver regeneration.^26^ There is growing evidence that extracellular matrix deposition promotes carcinogenesis through the phosphoinositide 3 kinase (PI3K) and mitogen-activated protein kinase (MAPK) signaling cascades.^27^ The contribution of chronic inflammation in HCC development has been well examined.^28^ Overall, we saw a significant reduction of fibrosis in both the rat DEN model and the mouse DEN+CDAHFD model using morphometric quantification of collagen deposition as well as the expression of several pro-fibrotic genes. Pro-Inflammatory cytokine expression was also decreased by pioglitazone in the DEN+CDAHFD mouse model. This effect was not seen in the rat DEN model. This might be due to the fact that the inflammatory insult is constant in the DEN+CDAHFD mouse model, but the inflammatory insult in the DEN model is given on a weekly basis. In the latter case the animals were sacrificed 1 week after the last DEN injection allowing time for the inflammatory response to subside on its own.

Pioglitazone targets PPARγ which belongs to a family of nuclear hormone receptors, with three different isoforms, PPAR alpha, gamma, and beta/delta.^29^ TZDs, such as pioglitazone, activate PPARγ which is predominately expressed in adipose tissue. Activated PPARγ mediates the release of adiponectin from adipocytes which has several important functions including improving insulin-sensitization and reducing inflammation.^30^ We observed that pioglitazone increased adiponectin release which activated AMPK in the liver leading to a downregulation of the MAPK pathways (mainly ERK/JNK/cJUN). These results were remarkably consistent in both the rat DEN and mouse DEN+CDAHFD models. In our NASH cirrhosis model, we also observed that CDAHFD activated Acetyl-CoA carboxylase (ACC), the rate-limiting step of fatty acid synthesis, and treatment with pioglitazone decreased ACC activation and steatosis as seen with the reduction in the NAS score and quantification of lipid vacuolization.

One limitation of this study is the use of only male animals. It is well documented that female rodents are relatively protected from both fibrosis progression and HCC development.^31, 32^ The gender-related incidence of HCC carcinogenesis has been reported to be largely due to the inhibitory effect of estrogen-dependent IL-6 production.^33^ Globally, HCC occurs more frequently in men than in women, with a male to female ratio ranging between 8:1 and 2:1.^34^ Women have also been shown to have less aggressive HCC tumors than men at initial diagnosis.^35^ However, underrepresentation of females in preclinical studies is a major concern with respect to better understanding the role of female biology on cancer development. Future endeavors are needed to better address gender discrepancy and develop female animal models of HCC.

In pre-diabetes or patients with type-2 diabetes and biopsy-proven NASH, long-term pioglitazone treatment resulted in a 51% resolution of NASH and improvement in fibrosis score as well as insulin sensitivity.^36^ More recent results suggest that pioglitazone reduces advanced fibrosis in NASH even in patients without diabetes.^37^ However, its use has been limited at least in part due to concerns about increased bladder cancer risk in patients who receive long-term pioglitazone treatment.^38^ These concerns may be overstated given the overall low risk of bladder cancer in patients with diabetes as well as the much greater beneficial effects of pioglitazone on cardiovascular disease and NASH.^39^ Our results in two animal models are consistent with the data from human studies demonstrating reduced fibrosis after pioglitazone treatment and suggest that pioglitazone should be resurrected as a treatment for NASH and even further should be investigated as an HCC chemopreventive strategy in prospective trials, especially given the approximately 3–13% annual incidence for HCC development in patients with NASH-related cirrhosis.^40^

## Conclusion

This study is one of the first to examine the effects of pioglitazone as a chemopreventive drug in animal models. Our results suggest that besides for its insulin sensitivity functions, pioglitazone reduces fibrosis and could be repurposed as an agent for HCC chemoprevention especially in the setting of NASH. Given its good safety profile and its frequent use, pioglitazone could be further explored in the setting of clinical trials in combination with other HCC chemopreventive agents.

## Electronic Supplementary Material

Supplementary Figure 1 Pioglitazone treatment improves bilirubin in the rat DEN model. Serum was collected and liver function tests were performed including A) aspartate aminotransferase (AST), B) alanine aminotransferase (ALT), C) Gamma-glutamyl transferase (GGT), D) alkaline phosphatase (ALP) and E) total bilirubin (TBL). # *p* < 0.05 and ## *p* < 0.01 compared to PBS. * *p* < 0.05 compared to DEN. (PPTX 156 kb)

Supplementary Figure 2 Pioglitazone treatment did not alter 8-hydroxydeoxyguanosine (8- OHdG) levels in the rat DEN model. Liver tissue levels of 8-OHdG levels were measured. # *p* < 0.05 compared to PBS. (PPTX 100 kb)
