# Supplementary figures and images for: Pioglitazone Reduces Hepatocellular Carcinoma Development in Two Rodent Models of Cirrhosis
Source: J Gastrointest Surg. 2018 Oct 26;23(1):101–11. doi: 10.1007/s11605-018-4004-6 (PMC6328630; doi:10.1007/s11605-018-4004-6)

## Slide 1
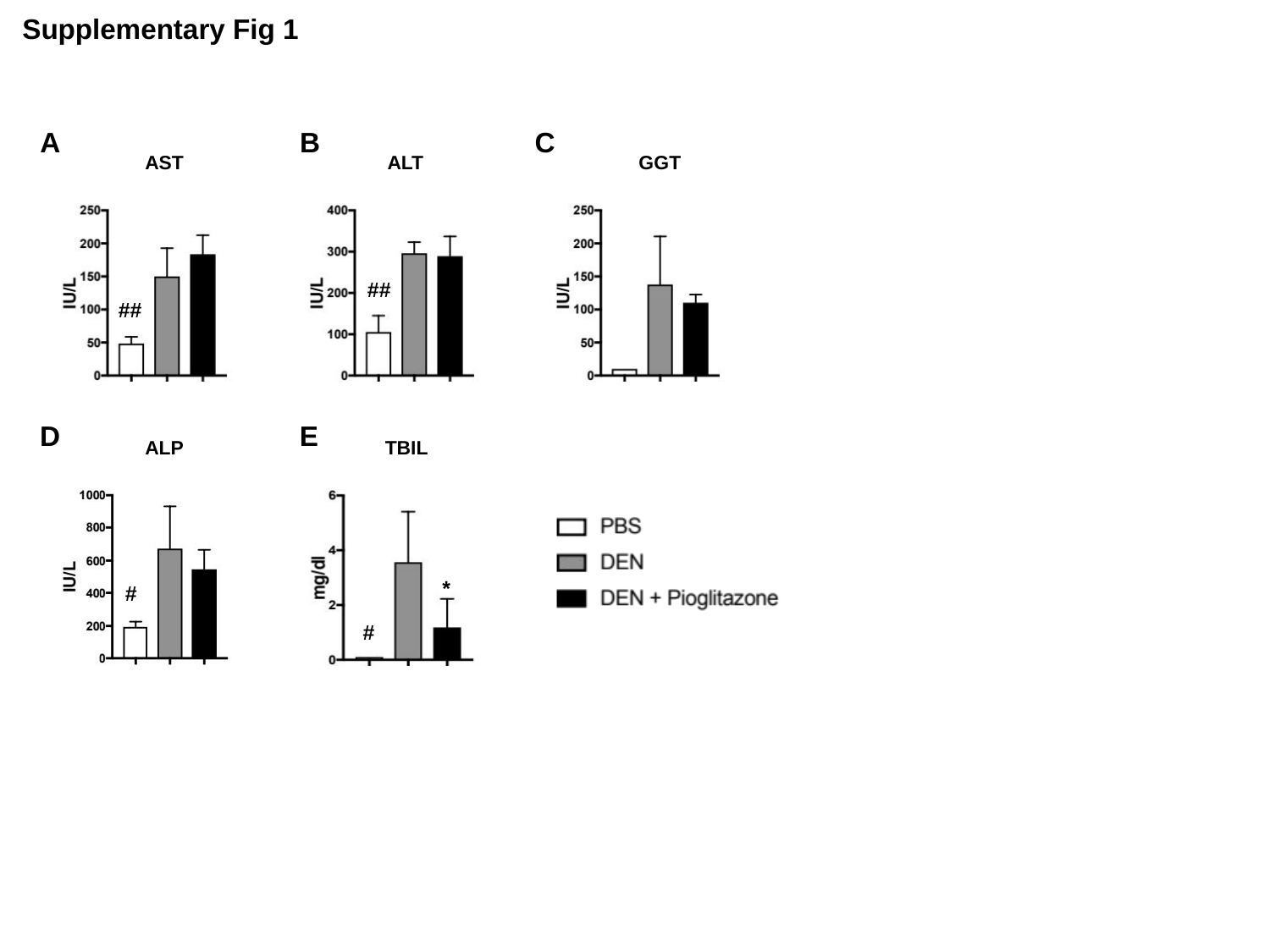

# Supplementary Fig 1
A
B
C
AST
ALT
GGT
##
##
D
E
ALP
TBIL
*
#
#

Supplement: Supplementary file 1 — Pioglitazone treatment improves bilirubin in the rat DEN model. Serum was collected and liver function tests were performed including A) aspartate aminotransferase (AST), B) alanine aminotransferase (ALT), C) Gamma-glutamyl transferase (GGT), D) alkaline phosphatase (ALP) and E) total bilirubin (TBL). # p < 0.05 and ## p < 0.01 compared to PBS. * p < 0.05 compared to DEN. (PPTX 156 kb) [file 11605_2018_4004_MOESM1_ESM.pptx]

## Slide 1
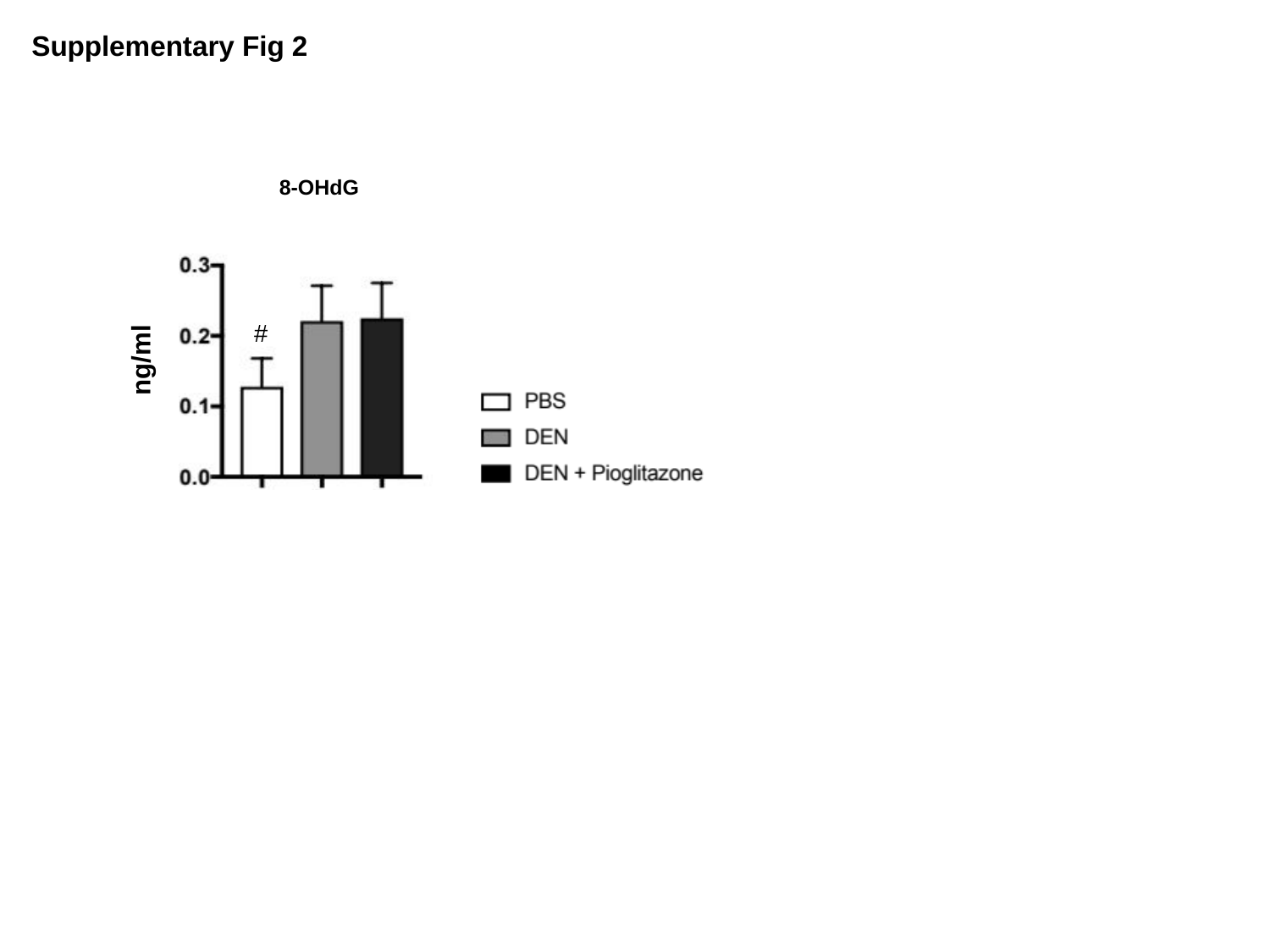

Supplementary Fig 2
8-OHdG
#
ng/ml

Supplement: Supplementary file 2 — Pioglitazone treatment did not alter 8-hydroxydeoxyguanosine (8- OHdG) levels in the rat DEN model. Liver tissue levels of 8-OHdG levels were measured. # p < 0.05 compared to PBS. (PPTX 100 kb) [file 11605_2018_4004_MOESM2_ESM.pptx]
